# Do women empowerment indicators predict receipt of quality antenatal care in Cameroon? Evidence from a nationwide survey

**DOI:** 10.1186/s12905-021-01487-y

**Published:** 2021-09-28

**Authors:** Edward Kwabena Ameyaw, Kwamena Sekyi Dickson, Kenneth Setorwu Adde, Obidimma Ezezika

**Affiliations:** 1 The Australian Centre for Public and Population Health Research, Faculty of Health, University of Technology Sydney, Sydney, NSW Australia; 2 Department of Population and Health, College of Humanities and Legal Studies, University of Cape Coast, Cape Coast, Ghana; 3 Department of Health and Society, University of Toronto Scarborough, Toronto, Canada

**Keywords:** Quality, Antenatal, Women, Reproductive health, Pregnancy, Cameroon

## Abstract

**Background:**

World Health Organisation (WHO) recommends quality antenatal care (ANC) for all pregnant women, as one of the strategies for achieving targets 3.1 and 3.2 of the sustainable development goals. Maternal mortality ratio remains high in Cameroon (782 maternal deaths per 100,000 live births). Extant literature suggest a positive association between women empowerment indicators and maternal healthcare utilisation in general. In Cameroon, this association has not received scholarly attention. To fill this knowledge gap, we investigated the association between women empowerment indicators and quality ANC in Cameroon.

**Methods:**

Data of 4615 women of reproductive age were analysed from the women’s file of the 2018 Cameroon Demographic and Health Survey. Quality ANC (measured by six indicators) was the outcome of interest. Binary Logistic Regression was conducted. All results of the Binary Logistic Regression analysis were presented as adjusted odds ratios (aORs) with 95% confidence intervals (CIs). All analyses were done using Stata version 14.

**Results:**

In all, 13.5% of the respondents received quality ANC. Women with low knowledge level (aOR = 0.66, CI 0.45, 0.98) had a lesser likelihood of receiving quality ANC compared to those with medium knowledge level. Women who highly approved wife beating (aOR = 0.54, CI 0.35, 0.83) had lesser odds of receiving quality ANC compared to those with low approval of wife beating.

**Conclusion:**

The study has pointed to the need for multifaceted approaches aimed at enhancing the knowledge base of women. The Ministry of Public Health should collaborate and intensify female’s reproductive health education. The study suggests that women advocacy and maternal healthcare interventions in Cameroon must strive to identify women who approve of wife beating and motivate them to disapprove all forms of violence.

## Introduction

Although the global maternal mortality rate has declined by 38% (from 342 to 211 deaths per 100,000 live births between 2000 and 2017), it remains miles away from reaching the desired 70 maternal deaths per 100,000 live births goal of the sustainable development goal 3 (SDG) [1]). Globally, the annual number of women who die as a result of pregnancy and childbirth has declined by 38% from 342 to 211 deaths per 100,000 live births between 2000 and 2017, nevertheless over 800 women are dying daily from pregnancy and childbirth-related complications [1]. Sub-Saharan African countries suffer the highest maternal mortality ratio of about 68% maternal deaths annually worldwide.

Inadequate utilisation of antenatal care (ANC) services is one of the key contributors to maternal mortality [2]. ANC enables for early detection and treatment of pregnancy-related complications as well as the prevention and management of concurrent diseases [3]. As such, quality ANC is prescribed by the WHO instead of just meeting the recommended timing and number of visits [4].

Several factors such as cost and distance among many others have shown to be barriers to ANC [5]. However, the lack of empowerment on the side of women may also negatively affect ANC visits and the quality of service they receive [3]. Blackstone [6] observed that women often lack decision making power to seek ANC services. Consequently, such decisions are mainly taken by their husbands or partners [7].

In Cameroon, women’s efforts are hardly recognised and women often lack developmental opportunities. Women are usually not consulted in decision making on critical issues that directly affect their reproductive lives and productivity at the household level [8]. A number of policies/laws in Cameroon discriminate against women and there are no legal reforms to enhance the protection of women empowerment. Sexual harassment at the workplace is immanent and not punishable by law [9]. Some women empowerment strategies are underway though. For instance, non-governmental organisations (NGOs) are working with women's groups to empower the women [8]. The government and the UN Women and the government (through the Ministry of Women's Empowerment and Family, Ministry of Economy, Planning and Spatial Planning and Ministry of Health) are advocating and supporting women’s political empowerment by offering technical assistance to women leaders and political parties [10].

Sexual and reproductive health studies have shown that increased women empowerment is associated with improvement in women’s health outcomes [See, 11–14]. Pratley [14] also observed that in 67 low and middle-income (LMIC) countries, women empowerment had a positive association with health service utilisation.

Although there has been a global decline in maternal mortality ratio, this is not the case for Cameroon [15] with a high ratio of 782 maternal deaths per 100,000 live births [16]. Consequently, studies have examined the determinants of maternal mortality [15–18] as well as factors that influence the utilisation of ANC [19–22] in Cameroon. However, to our best of knowledge, the role of women empowerment indicators in the utilisation of quality ANC has not been examined.

Pursuant to this, there is a need to fill this knowledge gap. This study, therefore, sets out to investigate the association between women empowerment indicators and receipt of quality ANC in Cameroon. Findings from this study could enlighten the government and all stakeholders to plan and design effective and fit for purpose quality ANC interventions and accelerate the nation’s prospects of achieving the SDG targets 3.1 and 3.2.

## Conceptual framework

**Fig. 1:**
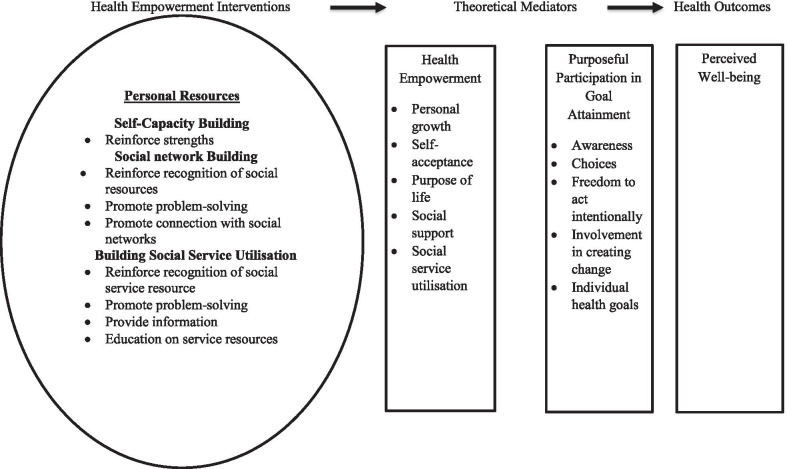
Health Intervention Framework. Source: Shearer and Reed [23]

The study is anchored in the Health Empowerment Intervention framework by Shearer and Reed [23]. The framework provides an explicit relationship between empowerment and health outcomes (see Fig. 1). The framework was developed as an approach to understanding mediators influencing health [23]. While health empowerment constitutes personal growth, self-acceptance, purposeful life, social support and social service utilisation; purposeful participation in goal attainment is concerned with awareness and choices made about prevailing health interventions, freedom to act intentionally as well as individual health goals [23, 24]. For the purpose of this study, empowerment of Cameroonian women is expected to translate into receipt of quality ANC, which can guarantee good or desirable birth outcome.

## Materials and methods

### Data

The data for the study was from the women's file of the 2018 Cameroon Demographic and Health Survey (CDHS). The Demographic and Health Survey (DHS) captures data on various aspects of demographic and health indicators. CDHS collected information on sexual activity, contraceptives, fertility, antenatal care, delivery, postnatal care, physical and sexual violence. The survey is a nationwide survey, with a representative sample of a total of 13,527 women interviewed from 11,986 households which were derived from 470 clusters (urban = 245 cluster and rural = 225 clusters). For the purpose of our present study, only women who gave birth within five years prior to the survey and went for antenatal care during their pregnancy were included [25]. The dataset is freely available for download at: https://dhsprogram.com/data/dataset/cameroon_Standard-DHS_2018.cfm?flag=0

### Definition of variables

#### Dependent variable

The dependent variable was quality ANC. Quality ANC, in this study, refers to the receipt of WHO recommended services at pregnancy. This definition for quality ANC was premised on literature [26–28] and in line with the definition of quality of care as proffered by Donabedian [29] i.e. the magnitude to which healthcare aligns with requirement for standard care. The elements of quality ANC consist of: 1. if the woman took fansider during pregnancy, 2. if she had tetanus injection before birth, 3. blood pressure taken during pregnancy, 4. urine sample taken during pregnancy, 5. iron supplements taken during pregnancy, and 6. blood sample taken during pregnancy. An index was created with scores ranging from 0 to 6. The score 0 to 5 (ranging from women without any of the services [0] up to those with 5 of the total services [5]) was labelled as “incomplete” and 6 was labelled as “complete” to denote women who had all the six services [26–28]. A dummy variable was generated with ‘0’ being women who had 0 to 5 of the quality ANC services and ‘1’ if women had all the six services of quality ANC.

#### Independent variable

The main independent variable was women empowerment indicators. The indicators used comprised; 1. Labour force participation (not working, working), 2. Acceptance of wife beating (neglect of child, going out without permission, arguing with husband/partner, burning of food, refusal to have sex with husband/partner), 3. Decision making power (measured by the person who makes decisions concerning respondent’s health care, house earning, household purchase, and visiting family members) 4. Knowledge level (measured by frequency of listening to radio, reading newspaper/magazine, watching television, and educational level). This is in conformity with the methods of previous authors [30, 31]. Other independent variables include age (15–19, 20–24, 25–29, 30–34, 35–39, 40–44, 45–49), place of residence (urban or rural), wealth status (poorest, poorer, middle, richer, richest), marital status (never in union, married, living with partner, widowed, divorced, separated), antenatal care visits (less than 4 or more than 4), and health insurance coverage (no or yes). The selection of these variables was premised on their association with quality ANC as reported by some previous research [26–28].

### Data analysis

Descriptive and inferential analyses were done. The descriptive analysis reported results on women empowerment indicators and the proportion of women who received quality ANC. Two models were used for the inferential analysis. Model 1 investigated the relationship between women empowerment indicators and quality ANC. Model 2 controlled for background characteristics. The Binary Logistic Regressions were conducted. All results of the Binary Logistic Regression analyses were presented as adjusted odds ratios (aORs) with 95% confidence intervals (CIs). Normative groups were chosen as reference categories in the models [32]. All analyses were done using Stata version 14.

### Ethics approval

This study benefited from publicly available data from DHS. Pre-approval was obtained from all participants prior to the survey. The DHS Program adheres to ethical standards to protect respondents' privacy. Inner-City Fund (ICF) International and the National Institute of Statistics (Institut Nationale de la Statistique) also ensure that the surveys are in line with the ethical requirements of Health and Human Services. No additional ethical approval is required because the data is secondary and publicly available. Details of the ethical standards are available on http://goo.gl/ny8T6X.

## Results

### Descriptive results

Nearly 14% of the respondents received quality ANC during pregnancy (see Table 1). A significant proportion of women with high knowledge level (15.5%), working (13.6%), high decision-making power (14.0%), and low approval of wife beating (14.4%) received quality ANC during pregnancy (see Table 1). Women aged 35–39 (15.5%), those in urban (16.0%), richest wealth status (19.2%), 4 or more ANC visits (15.9%), and covered with health insurance (17.6%) received quality ANC during pregnancy (see Table 1).

**Table 1:** Background characteristics and quality ANC received

Background characteristics	Frequency (n = 4615)	Percent (%)	Proportion of quality antenatal care received (%)
*Women empowerment*			
Knowledge level			
Low	528	11.4	6.8
Medium	2,253	48.8	13.4
High	1,834	39.7	15.5
Labour force participation			
Not working	1275	27.6	12.4
Working	3340	72.4	13.6
Decision making power			
Low	1985	43.0	13.3
Medium	855	18.5	13.0
High	1775	38.5	14.0
Approval of wife beating			
Low	3740	81.0	14.4
Medium	547	11.9	11.2
High	328	7.1	7.3
Age			
15–19	465	10.1	9.9
20–24	1057	22.9	13.2
25–29	1200	26.0	14.0
30–34	959	20.8	13.6
35–39	619	13.4	15.5
40–44	260	5.6	14.6
45–49	55	1.2	9.1
Place of residence			
Urban	2391	51.8	16.0
Rural	2224	48.2	10.8
Wealth status			
Poorest	540	11.7	7.0
Poorer	966	20.9	11.7
Middle	1159	25.1	11.4
Richer	1053	22.8	15.8
Richest	897	19.5	19.2
Marital status			
Never in union	719	15.6	15.7
Married	2495	54.1	12.8
Living with partner	1048	22.7	13.0
Widowed	74	1.6	18.9
Divorced	46	1.0	6.5
Separated	233		15.9
Antenatal care visit			
Less than 4	1123	24.3	6.0
More than 4	3492	75.7	15.9
Health insurance coverage			
No	4507	97.7	13.4
Yes	108	2.3	17.6
Total	4615	100	13.5

### Binary logistic regression on quality of ANC received among women in Cameroon

Two models were fitted, the first examined the relationship between the main explanatory variable-women empowerment indicators-and the quality of ANC. The second model examined all the background characteristics (women empowerment and other explanatory variables) and quality of ANC. Knowledge level, approval of wife beating, wealth status, and antenatal care visits were seen to have a significant association with the quality of ANC women received in Cameroon.

Women with low knowledge level (aOR = 0.66, CI 0.45, 0.98) had a lesser likelihood of receiving quality ANC compared to those with medium knowledge level (see Table 2). Women who were not working had lower odds of receiving quality ANC relative to those working (aOR = 0.83, CI 0.67, 1.02). However, higher odds were observed among those with medium decision-making power relative to those who had low decision-making power (aOR = 1.05, 1.41). Women with the high approval of wife beating (aOR = 0.54, CI 0.35, 0.83) had a lesser odd of receiving quality ANC compared to those with low approval of wife beating (see Table 2). Women with the richest wealth status (aOR = 1.49, CI 1.12, 1.99) were seen to have a higher likelihood of receiving quality ANC compared with those with middle wealth status. Also, women who made less than 4 antenatal visits (aOR = 0.40, CI 0.30, 0.52) had lesser odds of receiving quality ANC compared with those who have made 4 or more antenatal care visits (see Table 2).

**Table 2:** Binary logistic regression on quality of ANC received among women in Cameroon

Background characteristics	Model 1 Adjusted odds ratio (confidence interval)	Model 2 Adjusted odds ratio (confidence interval)
*Women empowerment*		
Knowledge level		
Low	0.48*** (0.58, 0.69)	0.66* (0.45, 0.98)
Medium	Ref	Ref
High	1.16 (0.97, 1.39)	0.91 (0.76, 1.12)
Labour force participation		
Not working	0.86 (0.70, 1.04)	0.83 (0.67, 1.02)
Working	Ref	Ref
Decision making power		
Low	Ref	Ref
Medium	0.97 (0.76, 1.23)	1.05 (0.77, 1.41)
High	0.97 (0.81, 1.18)	1.00 (0.76, 1.31)
Approval of wife beating		
Low	Ref	Ref
Medium	0.78 (0.58, 1.02)	0.85 (0.64, 1.14)
High	0.50** (0.33, 0.76)	0.54** (0.35, 0.83)
Age		
15–19		0.80 (0.56, 1.15)
20–24		0.98 (0.77, 1.26)
25–29		Ref
30–34		0.94 (0.73, 1.22)
35–39		1.11 (0.84, 1.48)
40–44		1.07 (0.72, 1.58)
45–49		0.66 (0.25, 1.71)
*Place of residence*		
Urban		Ref
Rural		0.93 (0.73, 1.17)
*Wealth status*		
Poorest		0.82 (0.53, 1.24)
Poorer		1.15 (0.87, 1.53)
Middle		Ref
Richer		1.30 (0.99, 1.69)
Richest		1.49** (1.12, 1.99)
*Marital status*		
Never in union		1.21 (0.88, 1.66)
Married		Ref
Living with partner		0.96 (0.76, 1.20)
Widowed		1.70 (0.89, 3.23)
Divorced		0.52 (0.16, 1.73)
Separated		1.20 (0.78, 1.84)
*Antenatal care visit*		
Less than 4		0.40*** (0.30, 0.52)
More than 4		Ref
*Health insurance coverage*		
No		Ref
Yes		1.02 (0.61, 1.71)

*p < 0.05; **p < 0.01; ***p < 0.001

*Ref* Reference category

## Discussion

The recent ANC recommendations by WHO emphasises the need for every pregnant woman to receive quality service throughout the pregnancy period but not only to achieve the recommended timing and number of visits [4]. Some evidence indicate that women empowerment indicators enhance maternal healthcare utilisation particularly ANC [30, 31]. In spite of these, no empirical study exists on the association between indicators of women empowerment and the proportion of women who receive quality ANC in Cameroon. We, therefore, investigated the association between indicators of women empowerment and quality ANC in the country. In all, 13.5% of the participants had quality ANC during their last pregnancy. Of the four empowerment indicators, two had a significant association with quality ANC: knowledge level and approval of wife beating. Specifically, women with low knowledge level and those who approved wife beating had lesser odds of quality ANC.

The findings on empowerment indicators and quality ANC affirm earlier reports on the positive association of these indicators and maternal healthcare utilisation [33–35]. The findings also affirm the position of the underlying conceptual framework of the study [23]. Several possibilities may account for these findings. With respect to knowledge, women with a high depth of knowledge are more probable to know the content of ANC and the need for them to receive the full content. Contrariwise, women who have low or limited knowledge may not know the array of services they are to receive and the services they should expect from healthcare providers during ANC visits. As posited by the Health Belief Model (HBM), knowledge possession about a service is a key determiner of one’s ability to utilise the service [36]. The study has pointed to the need for multifaceted approaches aimed at enhancing the knowledge base of women. It is, therefore, necessary for the Ministry of Health and the Ministry of Education to collaborate and intensify female health education.

Non-working women had lower odds of quality ANC, however, this was insignificant. This confirms the evidence that women who work have higher prospects of effective maternal healthcare utilisation [37, 38] and also reinforce the tenet of the Health Empowerment Intervention framework [23, 24]. Women who work are likely to have a wider social network and receive information from other women she meets at the work place. This may present an opportunity for them to learn about quality ANC and its importance and hence demand it during ANC visits, unlike those who do not work.

Women with medium decision-making capacity had higher odds of quality ANC relative to women with low decision-making capacity. This is anticipated considering the positive association between decision-making competencies and healthcare utilisaition [39–42]. It is anticipated for women who approved wife beating to have lesser odds of quality ANC. Literature indicates that women who approve of wife beating are mostly timid, poor, and have limited negotiation skills [43]. These characteristics may not propel them to inquire and insist on all the services that need to be received, even if they are fully aware of the services. This is an indication that women advocacy and maternal health interventions in Cameroon must strive to identify women who approve of wife beating and motivate them to disapprove of all forms of violence. This is critical because the study has subtly illustrated a positive association between women’s ability to denounce abuse (wife beating) and prospects of quality ANC in the Cameroonian context.

Approval of wife-beating is a key empowerment indicator that borders on the relationship between men and women. Well-being outcomes for women are often best in scenarios where the husband recognizes the wife’s power [44]. According to the theory of gender and power [45], three major social structures characterize the gendered relationships between men and women: the sexual division of labour, the sexual division of power, and the structure of cathexis. The third structure, cathexis, is particularly relevant to the issue of approval of wife-beating because it emphasizes the structure of affective attachments and social norms, characterized by the emotional and sexual attachments that women have with men [45]. This theory has been applied in similar contexts to explain the HIV risk among women [46]. Therefore, maternal programs, advocacy, and practitioners must consider these norms in their maternal education programs.

Women with the richest wealth status had a higher likelihood of quality ANC compared with those with middle wealth status. In spite of methodological variations, existing quantitative research on quality ANC illustrates that wealthier women have higher chances of quality ANC [26, 47–49]. Possible explanations have been advanced to expatiate the high tendency of quality ANC among women of high wealth standing. First, rich women are more likely to live in locations where quality care is immanent. Second, they can easily afford quality healthcare and thirdly they are likely to utilise quality healthcare [26] Besides, they are more likely to have a good relationship with health personnel. All these factors contribute positively towards access and utilisation of quality healthcare [26].

Women who made less than 4 antenatal visits were having lesser odds of quality ANC compared with those who had 4 or more antenatal care visits. This is consistent with previous evidence [26]. Women who obtain 4 or more ANC visits have more contact with healthcare providers and as a result, they are likely to receive much health education and the best of care [50, 51]. Invariably, 4 or more ANC attendance is an indication of a woman’s consciousness and commitment to her wellbeing in pregnancy.

### Strengths and limitations of the study

This study is the first in Cameroon to investigate quality ANC, as defined by the new recommendations of WHO, using the most recent national survey. The study followed rigorous and appropriate analytical procedures hence producing robust findings. It is, however, noteworthy that the survey is cross-sectional and as a result causal-inference is not possible. Besides, the outcome variable-quality ANC-was entirely based on recall, and therefore recall and social desirability biases are possible.

### Implications for practice and/or policy

The study has pointed to the need for multifaceted approaches aimed at enhancing women’s knowledge base. It is, therefore, necessary for the Ministry of Health and the Ministry of Education to collaborate and intensify female health education. The public’s sensitization about domestic violence and effective community-oriented education are critical in influencing attitudes and perceptions about domestic violence at the national level.

The government and programs working in the maternal health space should address the contextual factors limiting women’s access to ANC if we want to ensure their quality ANC experience. Women who made less than four antenatal visits had lesser odds of receiving quality ANC than those who have completed four or more antenatal care visits. The government should provide incentives and address contextual barriers that prevent pregnant women from complying with the new WHO recommendation of one ANC contact in the first trimester, two contacts in the second trimester (at 20 and 26 weeks of gestation), and five contacts in the third trimester (at 30, 34, 36, 38, and 40 weeks) [4]. Increasing compliance to these recommendations could dramatically increase the quality of ANC that women receive.

## Conclusion

The study has shown that only a few women in Cameroon receive quality ANC (13.5%). Quality ANC is associated with two key indicators of women empowerment, thus knowledge level and approval of wife beating. The study suggests that women advocacy and maternal healthcare interventions in Cameroon must strive to identify women who approve of wife beating and motivate them to disapprove of all forms of violence. It is time for the Ministry of Women's Empowerment to educate women through workshops and behavioural change communication strategies, for the women to appreciate the need for them to receive all essential ANC services. Family heads ought to remind and support women to ensure that they receive all essential pregnancy medications and services.

## Abbreviations

ANC: Antenatal care
CDHS: Cameroon demographic and health survey
CI: Confidence interval
DHS: Demographic and health survey
HBM: Health belief model
ICF: Inner-city fund
LMIC: Low and middle-income countries
NGO: Non-Governmental Organisation
SDG: Sustainable development goal
WHO: World Health Organization

## Declarations

### Availability of data and materials

All analysed data are freely available to the public through www.measuredhs.com.
